# Brief reviews

**DOI:** 10.1590/S1980-57642011DN05040014

**Published:** 2011

**Authors:** Sonia Maria Dozzi Brucki

**Affiliations:** 1 Behavioral and Cognitive Neurology Unit, Department of Neurology, and Cognitive Disorders Reference Center (CEREDIC). Hospital das Clínicas of the University of São Paulo School of Medicine, São Paulo SP, Brazil.

## MECHANISM OF AMYLOID REMOVAL IN PATIENTS WITH ALZHEIMER'S DISEASE TREATED WITH GANTENERUMAB.

**Ostrowitzki et al. Arch Neurol 2011. DOI 10.1001/archneurol.2011.1538**

Gantenerumab is a human anti-Aβantibody that binds specifically to Aβplaques; a human IgG1 with high affinity for fibrillar Aβ. The aims of this study was to elucidate the mechanism by which gantenerumab reduces the level of brain amyloid. Patients were randomized to receive 60 mg or 200 mg of gantenerumab (n=12 performed PIB-PET analysis) or placebo (n=4, were submitted to PIB-PET). Infusions were given every four weeks; patients receiving up to seven infusions were analyzed.

The mean (SD) percentage change from baseline over the specific PIB signal was 20.9% (15.6%) for the placebo group; 5.3% (19.7%) for 60 mg-group; and -14.9% (20.3%) for the 200 mg-group. This latter group differed from the placebo group.

The possible mechanism through which gantenerumab clears amyloid plaques is by Fc receptor/microglia-mediated phagocytosis, followed by lysosomal degradation as demonstrated for differentiated human macrophages.

## RELATIONSHIP BETWEEN EDUCATION AND DEMENTIA: AN UPDATED SYSTEMATIC REVIEW.

**Sharp & Gatz. Alzheimer Dis Assoc Disord 2011;25: 289-304.**

The authors provided a systematic literature review examining the relationship between dementia and education, from various databases from January 1985 to July 2010. Many observational and epidemiologic studies have revealed an association between these two entities. This review demonstrated that this relationship was not presented by all studies. Of eighty-eight studies analyzed, 51 studies reported a significant effect of lower education on risk for dementia, whereas 37 studies showed no significant relationship between lower education and a dementia outcome. A total of 61% of the 46 prevalence studies showed a significant effect of education, and 55% of the 42 incidence studies observed a similar effect.

Dividing studies into world regions revealed the same tendency for a significant effect of education where 19 out of 31 European studies, 17 out of 27 North American studies, 8 out of 18 Asian studies, and in 5 out of 9 Latin American studies showed this association.

It is interesting to observe that low educational levels varied greatly from study to study, ranging from illiterates to less than 15 years of education, whereas highest educational levels ranged from literate to more than 17 years.

## FREQUENCY OF ALZHEIMER'S DISEASE PATHOLOGY AT AUTOPSY IN PATIENTS WITH CLINICAL NORMAL PRESSURE HYDROCEPHALUS.

**Cabral et al. Alzheimers Dement 2011;7:509-513. doi: 10.1016/j.jalz.2010.12.008.**

NPH is characterized by gait disturbance, cognitive impairment, and urinary incontinence, besides ventriculomegaly out of proportion with cortical atrophy on neuroimaging exams. Treatment is based on placement of a CSF shunt. Identification of patients that will have a positive response remains a challenge. There is evidence of NPH patients with concomitant AD pathology.

Individuals selected from the Sun Health Research Institute (SHRI) Brain Donation Program (BDP) had their medical files assessed and NPH cases were identified. A total of 563 of the 761 cases autopsied had neuropathologic evidence of dementing illness; AD pathology was found in 331 (56%) cases, with an additional 94 cases having a secondary diagnosis of other dementia in addition to AD pathology.

Nine out of 563 (1.6%) cases had been clinically diagnosed with NPH during their lifetime. On review of autopsy reports, eight of these cases were found to have AD and one progressive supranuclear palsy. Concomitant neuropathologic diagnoses included one with DLB, three with cerebral white matter rarefaction, and two with argyrophilic grains.

## CLINICOPATHOLOGICAL CORRELATES OF BEHAVIORAL AND PSYCHOLOGICAL SYMPTOMS OF DEMENTIA.

**Casanova et al. Acta Neuropathol 2011;122:117-135.**

Psychiatric manifestations of dementia arise from specific dysfunction of brain systems. There is different progression in these symptoms in relation to cognitive deterioration.

Authors have presented a review concerning brain structures and BPSD. BPSD were grouped into psychiatric (hallucinations and delusions) and affective (depression and apathy) symptoms.

Neuropathological causes cannot explain all these symptoms. Authors made a list of possible explanations including environmental changes, neurochemical abnormalities, past psychiatric history, social and family history, and genetic susceptibility. The work constitutes an extensive review of this topic.

## DIAGNOSTIC CRITERIA FOR CORTICOBASAL SYNDROME: A COMPARATIVE STUDY.

**Mathew et al. J Neurol Neurosurg Psychiatr 2011; DOI: 10.1136/jnnp-2011-300875**

Authors compared three sets of well-known criteria (from Toronto, the Mayo Clinic and Cambridge) in a group of 40 consecutive patients with diagnosis of corticobasal syndrome (CBS).

CBS presents with variable neuropathology: AD, progressive supranuclear palsy and moreover, the classical pathology with tau-positive intracellular inclusions, cortical ballooned neurons, frontoparietal neuronal loss and gliosis. Nigral and basal ganglia degeneration could be seen in patients with progressive aphasia or frontotemporal dementia on presentation. The clinical syndrome has been proposed as CBS.

The study sample had a mean age of presentation of 67 years (7.34), the commonest features were disturbance of speech and language (95%) and limb apraxia (75%). Cognitive impairment was seen in all patients. At presentation, the greatest number of patients fulfilled the Cambridge criteria (67.5%), followed by the Toronto (47.5%). Thirty percent of patients satisfied all three criteria, and these patients were taken as a gold standard for clinical diagnosis. Subsequently, the authors examined the accuracy of diagnosis at presentation. The Cambridge criteria diagnosed the maximum number of patients on first visit (73.3%).

Under these criteria, patients must present insidious onset and gradual progression and no sustained response to levodopa treatment with two major criteria (focal or segmental myoclonus, asymmetrical dystonia, alien limb phenomenon, cortical sensory loss or dyscalculia, frontal executive dysfunction, visuospatial deficits) and two minor criteria (akinetic rigid syndrome, limb apraxia, speech and language impairment).

## NEUROPATHOLOGIC FEATURES ASSOCIATED WITH ALZHEIMER DISEASE DIAGNOSIS. AGE MATTERS.

**Middleton et al. Neurology 2011;77:1737-1744.**

This study discusses the precision of neuropathology in oldest old patients with clinical AD. The analyzed cases were drawn from the National Alzheimer's Coordinating Center (NACC) that had clinical cognitive diagnoses.

The patients with clinical AD diagnosis and age ≥85 years were female, had higher MMSE scores, and less frequent APOE E4 allele than those younger at death. For individuals with normal cognition, the frequency of neurofibrillary tangles (NFT) increased with age. When analyses were carried out and adjusted for age, sex, education, and APOE E4 status, those with a clinical diagnosis of AD were more likely to have all neuropathological features except macrovascular disease.

Neuropathologic features differentiated subjects with clinical AD diagnosis from those without more precisely among the younger group (70-74y) than among the oldest old (≥85y). AD pathology also differentiated persons with AD from those with normal cognition more precisely among the younger age groups than among the oldest old. Clinical diagnosis of AD was most strongly associated with NFT across all age groups, but this association was weaker among the oldest old.

## HIPPOCAMPAL SCLEROSIS IN ADVANCED AGE: CLINICAL AND PATHOLOGICAL FEATURES.

**Nelson et al. Brain 2011;134:1506-1518.**

Hippocampal sclerosis refers to neuronal cell loss and astrocytosis in the subiculum and hippocampus CA1 sector. It is associated with significant ante-mortem cognitive dysfunction but not with epilepsy (HS-Ageing).

The analysis was based on 1110 individuals from three autopsy series with ante-mortem longitudinal data: the University of Kentucky Alzheimer's Disease Centre, the Nun Study and the Georgia Centenarian Study. A total of 106 cases with autopsy-verified HS-Ageing and 1004 controls were included.

Whereas percentage of individuals with HS pathology increased progressively with age at death, the percentage of subjects with Braak stage V or VI neurofibrillary pathology and AD-type neuritic plaques tapered off after the age of 95 years.

Three hundred six cases were evaluated using TDP-43 immunohistochemistry (79 with HS and 227 without HS). A total of 89.9% of HS-Ageing was TDP-43 positive; the frequency of aberrant TDP-43 (cytoplasmic, neuritic or tangle-like) was much greater in HS individuals (OR 83.5, 95%CI 35.6-195.9).

There was no increased risk for HS pathology in cases with infarcts after controlling for patient age.

Cognitive profiles were analyzed dividing cases by AD pathology and HS pathology: verbal fluency test scores were higher, but word list delayed recall scores were lower in patients with HS-Ageing compared to patients with AD pathology only. There was however, some overlap between groups.

## PREVIOUS DESCRIPTION OF SUBTYPES OF ALZHEIMER'S PATHOLOGY

**Ricardo Nitrini^1^; Kaouê Lopes^1^; Sonia M.D. Brucki^1^**

We read with great interest the paper by Murray et al.^1^ which points to the existence of clinical and pathological subtypes of Alzheimer's disease (AD), mainly typical, hippocampal sparing and limbic-predominant subtypes of AD. There were several demographic, genetic and neuroimaging differences among the subtypes, but the outstanding difference was in age of onset. Limbic-predominant cases were older than the other two groups but nevertheless had a slower rate of progression and longer duration of disease symptoms at death than cases with the hippocampal sparing subtype. The authors stated that their review of the literature was limited to articles published in English since 1984.

However, they did not refer to the work by Delay & Brion^2^, who clearly divided cases into late onset dementia (at that time known as senile dementia), which have AD pathological findings that are less diffuse and with higher density in Ammon's horn (p. 186), and early onset AD in which the parieto-occipital and temporal regions, as well as Ammon's horn, are invariably severely involved by the pathological process (p.11). Delay & Brion also described "atypical" cases of AD that have more severe cortical involvement, mainly of the occipital, occipitoparietal or frontotemporal areas, which were either forms with diffuse involvement together with focal reinforcement or focal cortical forms (p. 90-91). These atypical cases are reminiscent of the hippocampal sparing cases described by Murray et al., and in both papers they predominate in early onset AD.

We feel these descriptions from former authors should be acknowledged and believe they lend further support to the findings of Murray et al.

^1^MD, Cognitive and Behavioral Neurology Unit, Clínicas Hospital, University of São Paulo Medical School.

**Ricardo Nitrini** - 1061, Verbo Divino St , ap 52a Tower 4 São Paulo, Brazil. Zip code: 04719-002. E-mail: rnitrini@uol.com.br, phone number: +551199094051
